# Surgical Interventions for the Treatment of Supracondylar Humerus Fractures in Children: Protocol of a Systematic Review

**DOI:** 10.2196/resprot.8343

**Published:** 2017-11-21

**Authors:** Oreste Lemos Carrazzone, João Carlos Belloti, Fabio Teruo Matsunaga, Nacime Salomão Barbachan Mansur, Marcelo Hide Matsumoto, Flavio Faloppa, Marcel Jun Sugawara Tamaoki

**Affiliations:** ^1^ Department of Orthopedics and Traumatology Escola Paulista de Medicina Universidade Federal de São Paulo São Paulo Brazil

**Keywords:** supracondylar humerus fracture, children, surgical treatment, systematic review

## Abstract

**Background:**

The treatment of supracondylar humerus fracture in children (SHFC) is associated with complications such as functional deficit, residual deformity, and iatrogenic neurological damage. The standard treatment is closed reduction and percutaneous Kirschner wire fixation with different configurations. Despite this fact, there is still no consensus on the most effective technique for the treatment of these fractures.

**Objective:**

The aim of this systematic review will be to evaluate the effect of surgical interventions on the treatment of Gartland type II and III SHFC by assessing function, complications, and error as primary outcomes. Clinical outcomes such as range of motion and pain and radiographic outcomes will also be judged.

**Methods:**

A systematic review of randomized controlled trials or quasi-randomized controlled trials evaluating the surgical treatment of SHFC will be carried out in the Cochrane Central Register of Controlled Trials, PubMed, Literatura Latino-Americana e do Caribe em Ciências da Saúde, and Excerpta Medica Database. The search will also occur at ongoing and recently completed clinical trials in selected databases. Data management and extraction will be performed using a data withdrawal form and by analyzing the following: study method characteristics, participant characteristics, intervention characteristics, results, methodological domains, and risk of bias. To assess the risk of bias of the included trials, the Cochrane Risk of Bias Tool will be used. Dichotomous outcome data will be analyzed as risk ratios, and continuous outcome data will be expressed as mean differences, both with 95% confidence intervals. Also, whenever possible, subgroup analysis, sensitivity analysis, and assessment of heterogeneity will be performed.

**Results:**

Following the publication of this protocol, searches will be run and included studies will be deeply analyzed. We hope to obtain final results in the next few months and have the final paper published by the end of 2018. This study was funded by a government-based noncommercial agency, Fundação de Amparo à Pesquisa do Estado de São Paulo (FAPESP).

**Conclusions:**

This study may provide surgical treatment effects evidence for SHFC. The results will assist clinical practice by demonstrating the effectiveness and potential complications of these interventions and might serve as a reference for future clinical trials on the topic.

**Trial Registration:**

PROSPERO CRD42014009304; https://www.crd.york.ac.uk/prospero/display_record.php?RecordID=9304 (Archived by WebCite at http://www.webcitation.org/6usiDHzD7)

## Introduction

### Overview

Supracondylar humerus fractures are the most frequent elbow fracture in the pediatric population [1,2]. They are associated with significant complications, such as neurovascular injuries and malunion [3,4,5]. Cubitus varus is the most common residual deformity from supracondylar fractures [6-10], usually due to malreductions [7,9,11-13] that promote distal fragment rotational displacement [14]. Other causes are medial column comminution developing reduction loss during the follow-up [15] and growth arrests caused by physeal injuries [16-21].

The most common mechanism of injury is a fall to the extended arm, this corresponding to over 95% of cases [22]. Another mechanism is direct trauma to the posterior region of the flexed elbow, generating an anterior deviation of the distal fragment of the fracture. The most widely used classification for these fractures is described by Gartland (ie, type I: undisplaced or minimally displaced, type II: displaced, but with intact posterior cortex, and type III: completely displaced with no cortical contact) [23]. Nerve damage associated with trauma occurs in about 11.3% of patients and vascular lesions in less than 1% of these fractures. The ulnar nerve is the most commonly injured nerve in flexion fractures. It may also be iatrogenically damaged during percutaneous fixation of the medial column of the distal humerus [24]. The objective of surgical treatment of displaced and unstable fractures (Gartland types II and III) is to obtain a stable reduction, prevent neurovascular injuries, avoid compartment syndrome [3,4,5,25], and lower the risk of residual deformities, particularly cubitus varus. The standard treatment is closed reduction and percutaneous Kirschner wire (K-wire) fixation [26]. This fixation can be achieved by different configurations; the most commonly used are 2 crossed wires and fixation in the lateral column.

Biomechanics trials [27] show that the disposition of 2 crossed K-wires, 1 in the lateral column of the distal end of the humerus and the other in its medial end [28,29], is the configuration that provides the greatest stability when fixating these fractures. However, there is a risk of iatrogenic injury, mainly in the ulnar nerve [30,31] due to its close anatomical relationship with the posterior surface of the medial epicondyle. To minimize this complication, alternatively the fractures can be fixated with parallel or divergent K-wires only at the lateral column of the humerus [32]. This configuration is less stable [33,34] and could lead to residual deformities such as cubitus varus. In sum, there is still no consensus on the most effective technique for the treatment of displaced supracondylar humerus fractures [35].

### Objectives

The aim of this study is to evaluate the effectiveness of surgical interventions for the treatment of Gartland type II and III supracondylar humerus fractures in children and their complications. Therefore, we developed the following PICOS (patient, problem, or population; intervention; comparison, control, or comparator; outcome; study design) strategy: the population will consist of individuals with immature skeletons and acute history of displaced supracondylar fracture (Gartland type II or III); the intervention will be the surgery performed with insertion of 2 parallel or divergent K-wires at the lateral column of the humerus; the control will be patients submitted to the surgery with the insertion of 2 crossed K-wires (one at the lateral column of the humerus distal end and the other at its the medial end); the outcome will be the function assessed by validated scores and the complications; and the study design will be systematic review of the literature.

Our hypothesis is that fracture fixation with 2 K-wires at the lateral column will properly restore elbow function and minimize the risk of iatrogenic injury to the ulnar nerve.

## Methods

### Criteria for Considering Studies for This Review: Types of Studies, Participants, and Interventions

This will be a systematic review of randomized controlled trials or quasi-randomized controlled trials on the surgical treatment of supracondylar humerus fractures in children, without restrictions to language, status, or year of publication.

This protocol was developed according to the criteria described in the Cochrane Handbook of Interventions Reviews [36] and the Preferred Reporting Items for Systematic Review and Meta-Analysis protocols [37].

The participants include children (immature skeleton) with displaced supracondylar fracture (Gartland type II or III) and a history of acute trauma (less than 2 weeks), no prior deformity of the studied elbow, and an absence of concomitant fractures in the ipsilateral limb.

The 2 surgical interventions described for the treatment of acute supracondylar fractures are insertion of 2 crossed K-wires, 1 at the lateral column of the distal end of the humerus and the other at its medial end, and insertion of 2 parallel or divergent K-wires only at the lateral column of the humerus.

### Ethics Approval

This study was registered and authorized by the Research Ethics Committee of Universidade Federal de São Paulo (protocol number 108538/2015).

### Primary Outcomes

The primary outcomes will be the functional results, complications, and errors resulting from interventions. A validated elbow function score using the method by Flynn et al [12], the Disabilities of the Arm, Shoulder, and Hand Questionnaire [38], or the Mayo Elbow Performance Score [39] will measure function.

Complications will be interpreted according to type, severity, and date of occurrence. Some examples of complications are nerve iatrogenic injuries (ulnar, radial, or median), infections caused by the introduction of the K-wire, compartment syndrome, and deformities (cubitus varus or valgus). The complication’s severity will be classified as major (eg, permanent neurological damage with return to function through surgical procedure, deep infection requiring surgical intervention) or minor (eg, transient neurological injury with spontaneous return of function, superficial infection requiring antibiotic therapy). The date of the occurrence of complications will be dated as early (occurring up to 4 weeks after surgery or until the removal of wires) or late (more than 4 weeks after surgery or after wire removal).

Any surgical procedure other than the preestablished treatment protocol will be classified as an error based on the principle of intention to treat. An example of such a procedure is the inclusion of a third medial or lateral K-wire to add stability to the construct.

### Secondary Outcomes

Secondary outcomes will be divided into clinical and radiographic outcomes. Clinical outcomes will assess the variation in elbow range of motion and pain. The variation in range of motion is the change in the arc of total elbow movement in the late postoperative period compared to the contralateral limb, measured in degrees. Pain will be estimated using a validated instrument [40,41].

The radiographic outcomes will appraise variation of the carrying angle [42-44], variation of Baumann’s angle [45,46], and heterotopic ossification presence. The carrying angle is determined by drawing 2 lines in anteroposterior elbow radiographs, 1 along the humeral shaft long axis and the other along the ulna long axis. Baumann’s angle is obtained by the intersection of 2 lines, 1 parallel to the humeral shaft and the other parallel to the epiphyseal line of the lateral condyle drawn in elbow anteroposterior radiographs. This angle should be approximately 72°. The analysis of the carrying angle and Baumann’s angle will consider variations in these angles in the immediate postoperative radiograph compared to the follow-up radiograph (removal of synthesis material or 3 months postoperatively). In both angles, the difference to the contralateral side will be calculated. The heterotopic ossification will be judged dichotomously (positive or negative).

### Search Strategy

The searches will be carried out in the Cochrane Library, PubMed, Excerpta Medica Database, and *Literatura Latino-Americana e do Caribe em Ciências da Saúde* (LILACS). In addition, ongoing and recently completed clinical trial protocols will be searched in the ISRCTN Registry (www.isrctn.com), International Clinical Trials Registry Platform, ClinicalTrials.gov, Cochrane Central Register of Controlled Trials, LILACS , and *Plataforma Brasil*. There will be no restrictions to language or publication status. Subject headings and their search synonyms will be used. In MEDLINE (PubMed), the first 2 phases of the Cochrane highly sensitive search strategy for reports of randomized controlled trials [47] will be combined with the subject-specific search: “supracondylar,” “humerus,” “fractures,” and “surgery.”

### Selection of Trials

Two authors will independently select and evaluate potentially eligible trials for inclusion in the review through the title and abstract. All potentially eligible trials will be reviewed in their entirety, including those that cannot be identified based on title or abstract. Any differences will be resolved through discussion and, when necessary, a third author will solve the conflict.

### Data Management and Extraction

Two authors will extract the following data using a data extraction form: (1) study methodology characteristics, including study design and duration, whether the protocol was published before patient recruitment, possible funding sources, and trial registration; (2) participant characteristics, including location, number of recruited participants, number of evaluated participants, inclusion criteria, exclusion criteria, age, and injury classification; (3) intervention characteristics, including intervention duration, surgery type, and any complications; (4) results, including follow-up time and follow-up loss; and (5) methodological domains and bias risk assessment as described below. Both researchers will enter their data on forms.

### Included Trials Bias Risk Assessment

The bias risk of the included trials will be evaluated independently by 2 authors using the Cochrane Risk of Bias tool [48]. This will be done using the following criteria: random sequence generation, allocation concealment, participant blinding, outcome assessment blinding, incomplete outcome data, selective reporting, and other biases (eg, financial incentives use, population imbalance between groups). Each of these criterion will be explicitly judged and classified as low risk of bias, high risk of bias, or unclear risk of bias. Disagreements between the authors about the risk of bias for each of the domains will be resolved by consensus.

### Statistical Analysis

The Review Manager (Cochrane Collaboration) tool will be used for the statistical analysis. The dichotomous data will be analyzed by calculating the relative risk with a 95% confidence interval. The Mantel-Haenszel statistical method will be used, and continuous data will be analyzed using mean and standard deviation.

When the data of 2 or more trials is derived from the same validated assessment tool (with the same units of measurement), the data will be grouped as mean difference. The statistical method will be the inverse variance method. However, when primary trials express the same variables in different instruments or different units of measurement, standardized mean difference will be applied.

### Unit of Analysis

The randomization unit in the included trials usually is the individual participant. Exceptionally, as in case of trials including children with bilateral fractures, the data is evaluated per fracture, instead the individual.

### Dealing With Missing Data

In order to include all participants randomized to an intervention, intention-to-treat analysis will be performed. In case of inadequate information regarding the estimated effects, number of patients, mean, uncertainty measures (standard deviation or error), or number of events, the authors of the primary studies will be contacted.

### Assessment of Heterogeneity

The heterogeneity of the effects between the included trials will be visually analyzed using forest plots and the I^2^ test. Heterogeneity is considered significant when I^2^ is greater than 50%.

### Data Synthesis

Where appropriate, the authors will group the results of both surgical techniques and compare them using the fixed or random effects model with 95% confidence interval.

### Bias in the Meta-Analysis

If more than 10 trials are available, the publication bias and effect bias of small trials will be assessed visually with the funnel plot when possible. If asymmetry is found upon visual inspection, we will attempt to identify the reason.

### Confidence in Cumulative Evidence

The Grading of Recommendations Assessment, Development, and Evaluation tool (www.gradepro.org) will be applied to describe the quality of the evidence and the strength of the recommendations.

## Results

Following the publication of this protocol, searches will be run and included studies will be deeply analyzed. We hope to obtain final results in the next few months and have the final paper published by the end of 2018. This study was funded by a government-based noncommercial agency, *Fundação de Amparo à Pesquisa do Estado de São Paulo* (FAPESP).

## Discussion

Supracondylar humerus fracture fixation with crossed wires (medial and lateral) and fixation with 2 lateral wires can provide satisfactory results, although the complications inherent to these methods are not well established [49]. A complication reported is ulnar nerve injury due to medial K-wire insertion during the surgery, possibly by its proximity to the medial epicondyle. Conversely, inserting the wires only in the lateral column can reduce stability, leading to distal humerus malunion [50,51] despite all patients having their affected limb immobilized after surgery. Both neurological damage and precarious stability can cause patient limbs functional deficit. Also, assessment of residual varus deformity should be performed, as we believe that its main cause is poorly executed reduction during surgical procedure.

In the literature, we find few systematic reviews on the topic and, in most cases, these reviews include trials with low levels of evidence such as case series and nonrandomized clinical trials [52-57]. We did not find reviews with prior publication protocol, something that increases the chance of bias in the analysis. The last review on the subject was published a few years ago [58]. Since then, new trials have been published and should be included in our study.

We also observed that, in these revisions, the primary outcome was the iatrogenic injury to the ulnar nerve, but we believe that function and complications are the foremost outcomes to be assessed, given that most ulnar nerve injuries are transient and do not require a new intervention. Therefore, further evaluation of the literature on the supracondylar fracture surgical treatment with better methodological quality should be carried out covering functional, clinical, and radiographic outcomes as well as complications and treatment errors.

The main limitation is expected to be the difficulty finding trials with adequate sample size as well as the lack of standardization in the evaluation methods of the results. The results of this study may provide support and scientific evidence for decision making in orthopedic clinical practice regarding displaced supracondylar fractures of the distal humerus in children, serving as a guide for future trials with better methodological quality.

## Appendix

Multimedia Appendix 1 Peer-review reports.

## Abbreviations

FAPESP: Fundação de Amparo à Pesquisa do Estado de São Paulo
K-wire: Kirschner wire
LILACS: Literatura Latino-Americana e do Caribe em Ciências da Saúde
PICOS: patient, problem, or population; intervention; comparison, control, or comparator; outcome; study design
