# Supplementary material for: Surgical Interventions for the Treatment of Supracondylar Humerus Fractures in Children: Protocol of a Systematic Review
Source: JMIR Res Protoc. 2017 Nov 21;6(11):e232. doi: 10.2196/resprot.8343 (PMC5717449; doi:10.2196/resprot.8343)
Supplement: Multimedia Appendix 1 [file resprot_v6i11e232_app1.pdf]

Processo

Identificação do Processo

**Número do Processo**	2015/13159-3 - Projeto de Pesquisa - Regular
**Situação**	Encerrado
**Grupo de Financiamento**	Auxílio à Pesquisa
**Linha de Fomento**	Programas Regulares / Auxílios a Pesquisa / Projeto de Pesquisa / Projeto de Pesquisa - Regular - Fluxo Contínuo
**Beneficiário**	Marcel Jun Sugawara Tamaoki
**Responsável**	Marcel Jun Sugawara Tamaoki
**Data Início**	01/04/2016
**Duração**	12 mês(es)

**Instituição de Pesquisa/Empresa**	Escola Paulista de Medicina/EPM/UNIFESP
**Departamento**	Ortopedia e Traumatologia
**Data de Abertura**	25/06/2015

Projeto - Identificação

Título em Português

Efetividade no tratamento das Fraturas Supracondiliana e Luxações do Ombro

Título em Inglês

Interventions for treating supracondylar fractures and shoulder dislocation: systematic reviews.

Classificação

**Grande Área**	Ciências da Saúde
**Área**	Medicina
**Sub-área**	Outra Subárea Medicina
**Especialidade**	Ortopedia

**Palavras-chave**	Cirurgia do Ombro e Cotovelo, Estudos secundários, medicina baseada em evidências, Membro Superior, Ortopedia e Traumatologia, revisão sistemática/metanálise

Projeto - Instituições

Instituição de Pesquisa/Empresa Principal

**Nome**	Escola Paulista de Medicina/EPM/UNIFESP

Projeto - Pessoas Envolvidas

Equipe

Nome	Função	Horas Semanais Dedicadas ao Projeto	Vigência	Vínculo Principal

Marcel Jun Sugawara Tamaoki	Pesquisador Responsável *	30	01/04/2016 a 31/03/2017	Escola Paulista de Medicina/EPM/UNIFESP
Fabio Teruo Matsunaga	Apoio Técnico	30	01/04/2016 a 31/03/2017	
Flavio Faloppa	Apoio Técnico	10	01/04/2016 a 31/03/2017	Escola Paulista de Medicina/EPM/UNIFESP
Joao Carlos Belloti	Apoio Técnico	10	01/04/2016 a 31/03/2017	Escola Paulista de Medicina/EPM/UNIFESP
João Baptista Gomes dos Santos	Apoio Técnico	10	01/04/2016 a 31/03/2017	Escola Paulista de Medicina/EPM/UNIFESP
MIGUEL SABINO NETO	Apoio Técnico	10	01/04/2016 a 31/03/2017	Escola Paulista de Medicina/EPM/UNIFESP
ORESTE LEMOS CARRAZZONE	Apoio Técnico	30	01/04/2016 a 31/03/2017	
Paulo Henrique Schmidt Lara	Apoio Técnico	10	01/04/2016 a 31/03/2017	

* Com Benefício Complementar

Projeto - Descrição

Resumo em Português

A prática da medicina baseada em evidências requer o uso consciencioso da melhor evidência para a tomada de decisão em relação à saúde dos pacientes. Neste contexto, as Revisões Sistemáticas têm um papel importantíssimo. As revisões possibilitam realizar uma avaliação e síntese dos artigos científicos publicados na literatura científica. O presente proposta tem como finalidade o estudo do tratamento de duas das afecções ortopédicas mais prevalentes : as fraturas supracondilianas e as luxações do ombro. As fraturas supracondilianas são muito prevalentes e tem alto risco de complicações como deficiências neurovasculares, síndrome compartimental e consolidação viciosa. Já as luxações do ombro, representam quase 50% de todas as luxações articulares, e tem sido relatadas como sendo mais comum do que todas as demais luxações diartrodiais em conjunto. A incidência estimada da luxação da articulação do ombro nos Estados Unidos é de 23,9 para cada 100.000 habitantes ao ano. Diante da importância e por não haver evidência definitiva sobre o melhor tratamento para estas duas afecções decidimos desenvolver esta proposta. Portanto o objetivo específico desta proposta é avaliar a efetividade do tratamento para duas condições de extrema importância, as fraturas supracondilianas e as luxações do ombro, por meio de uma revisão sistemática de ensaios clínicos randomizados.

Resumo em Inglês

The practice of evidence-based medicine requires the conscientious use of the best evidence for decision-making regarding the health of patients. Systematic Reviews have an important role in this context. They allow conduct an assessment and synthesis of scientific articles. This proposal aims to asses the treatment of two of the most prevalent orthopaedic conditions: supracondylar fractures and shoulder dislocations. Supracondylar fractures are very frequent and have high risk of complications such as neurovascular injuries, compartment syndrome and malunion. Shoulder dislocations, represent nearly 50% of all joint dislocations, and has been reported to be more common than all other diarthrodial dislocations together. The estimated incidence of shoulder joint dislocation in the United States is 23.9 per 100,000 inhabitants per year. Since there is not a definitive evidence of the best treatment for these conditions we decided to develop this study. Therefore the specific purpose of this proposal is to evaluate the effectiveness of treatment for both conditions of extreme importance, supracondylar fractures and shoulder dislocations by a systematic review of randomised clinical trials.

Objetivos

O presente proposta tem como finalidade o estudo da efetividade do tratamento de duas das afecções ortopédicas mais prevalentes : as fraturas supracondilianas e as luxações do ombro.

Resultados Previstos

Espera-se com a atual proposta: - produção de evidências para tomada de decisão clínica, em específico para estas duas doenças altamente prevalentes no nosso meio, e que não tem um método padrão ouro para seus respectivos tratamentos. - orientação de como conduzir um estudo com a melhor qualidade metodológica possível para responder as pergunta clínica estudada, já que, em geral, as revisões sistemáticas pode chegar a dois desfechos ao seu final: evidência que um método é superior ou igual ao outro, ou não chegar a evidência conclusiva devido a falta de estudos primários. E neste caso, as revisões sistemática demonstra as falhas e melhor forma de conduzir um ensaio clínico randomizado. - atualização das revisões. Isso ocorre periodicamente, assim que novos estudos primários são publicadas, e caso seja necessário, incluiremos estes estudos em futuras atualizações.

O produto objeto do projeto é patenteável?

Não

R$ / US$ - Orçamento

Orçamento

**Benefícios**	**Valor (R$)**	**Valor (US$)**
Capital		
Material Permanente	3.005,10	0,00
Custeio		
Despesas de Transporte	0,00	0,00
Diárias	0,00	0,00
Material de Consumo	643,10	0,00
Serviços de Terceiros	0,00	249,95
Reserva Técnica - Benefícios Complementares	8.000,00	0,00
Reserva Técnica - Custo de Infraestrutura Direta do Projeto	697,20	0,00
Provisão para Importação	0,00	37,49
Outros	0,00	0,00
TOTAL	12.345,40	287,44
Bolsas		
Participação em Curso	0,00	0,00
Treinamento Técnico	0,00	0,00
TOTAL	0,00	0,00
TOTAL GERAL	12.345,40	287,44

Quotas de Bolsa

Modalidade / Categoria	Carga Horária	Duração (Meses)	Quantidade

Nenhuma quota solicitada.

R$ / US$ - Orçamento - Detalhamento

Material Permanente - Nacional

**Origem**	Brasil
**Quantidade**	1
**Classificação**	Microcomputador e componentes
**Descrição**	HP Pavilion All in One 23-P100 Intel Core i5-4690T 2.5 GHz 8192 MB 1024 GB
**Fabricado no Brasil**	Sim
**Valor Unitário**	3.005,10
**Valor Total**	3.005,10
**Justificativa**	Uso para pesquisa em bases eletronicas, banco de dados, estatística, comunicação entre os pesquisadores e elaboração do manuscrito.

Material de Consumo - Nacional

**Origem**	Brasil
**Classificação**	Material de Consumo
**Descrição**	Seagate Expansion STBV3000100 3072 gb EXTERNO
**Valor**	643,10
**Justificativa**	Amazenamento dos dados seguro, backup dos dados da pesquisa

Serviços de Terceiros - Importado

**Origem**	Exterior
**Quantidade**	1
**Classificação**	Serviço de Terceiros
**Descrição**	EndNote X7
**Moeda de Origem**	US$
**Valor Unitário**	249,95
**Taxa de Câmbio (US$)**	1,0000000
**Valor Total**	249,95
**Justificativa**	Essencial para organização e elaboração da revisão sistemática.

Reserva Técnica - Benefícios Complementares

	Nome	Papel	Valor	Vigência
**Beneficiados**	Marcel Jun Sugawara Tamaoki	Pesquisador Responsável	8.000,00	01/04/2016 a 31/03/2017

**Moeda**	R$
**Valor Unitário** (anual)	8.000,00
**Data de Referência**	21/03/2016
**Valor do Benefício Complementar**	8.000,00

Reserva Técnica - Custo de Infraestrutura Direta do Projeto

**Percentual para Reserva Técnica (País)**	15,00 %
**Percentual para Reserva Técnica (Exterior)**	15,00 %
**Dólar FAPESP**	4,00
**Valor Aumentado**	0,00
**Valor Diminuído**	0,00
**Valor da Reserva Técnica (R$)**	697,20
**Valor da Reserva Técnica (US$)**	0,00

Provisão para Importação

**Percentual para Provisão para Importação**	15,00 %
**Valor da Provisão para Importação (US$)**	37,49

R$ / US$ - Outras Fontes

Outras Fontes

Nenhuma outra fonte encontrada.

Documentos

Download de Todos os Documentos

1.1 Documentos Anexados na Proposta Atual (Reconsideração 003 submetida em 11/02/2016)

Tipo de Documento	Etapa Exigida	Arquivo	Data de Anexação	Arquivo Convertido
Formulário de		Formulario_de_Solicitacao_de_Reconsideracao-		

solicitação de reconsideração	Análise	SAGe v2.docx	11/02/2016	
Cópia de documento de identificação do beneficiário	Contratação	rg.pdf	22/03/2016	

1.2 Outros Documentos Anexados na Proposta Atual (Reconsideração 003 submetida em 11/02/2016)

Nenhum documento associado.

1.3 Documentos Anexados pela FAPESP na Proposta Atual (Reconsideração 003 submetida em 11/02/2016)

Nenhum documento associado.

2.1 Documentos Anexados até a Proposta Anterior (Reconsideração 002 submetida em 21/12/2015)

Tipo de Documento	Etapa Exigida	Arquivo	Data de Anexação	Arquivo Convertido
Anexo II - Informação sobre infraestrutura institucional necessária	Análise	termoII2.pdf	28/05/2015	
Formulário de solicitação de reconsideração	Análise	formulario_de_solicitacao_de_reconsideracao-SAGe 2015 r.docx	05/10/2015	
Manifestação do dirigente da instituição	Análise	JUN1.pdf	28/05/2015	
Orçamentos dos fornecedores/representantes autorizados	Análise	Orcamento fapesp 2015 v1.pdf	05/10/2015	
Orçamentos dos fornecedores/representantes autorizados.	Análise	cotações 26615.pdf	24/06/2015	
Parque de equipamentos	Análise	Descrição do Parque de Equipamentos científicos da.docx	28/05/2015	
Planos de atividades individuais para cada bolsa de treinamento técnico e/ou participação	Análise	Não se Aplica		
Projeto de pesquisa (auxílio)	Análise	Protocolo revisoes sistematicas 2015 v5.pdf	25/06/2015	
Resultados de auxílios anteriores	Análise	Não se Aplica		
Súmula curricular de cada um dos pesquisadores associados	Análise	Não se Aplica		
Súmula curricular do beneficiário	Análise	Súmula Curricular ABRIL 2015.pdf	25/06/2015	

2.2 Outros Documentos Anexados até a Proposta Anterior (Reconsideração 002 submetida em 21/12/2015)

Arquivo	Data de Anexação	Arquivo Convertido
TermoII.pdf	28/05/2015	

2.3 Documentos Anexados pela FAPESP até a Proposta Anterior (Reconsideração 002 submetida em 21/12/2015)

Nenhum documento associado.

Observações / Manifestações

Observações

Pedimos desculpas, pois na ultima proposta não foi anexado o formulário de reconsideração atualizado e sim o da versão anterior.

Histórico de Eventos

Histórico de Eventos

Descrição	Data
Processo Encerrado (Parte Científica e Administrativa)	15/09/2017
Resultado de Despacho Científico Divulgado - Relatório Científico 1	30/08/2017
Emissão de Despacho Científico Concluída - Relatório Científico 1	30/08/2017
Despacho Científico Iniciado - Relatório Científico 1	30/08/2017
Recomendação da Coordenação de Área/Programa Concluída - Relatório Científico 1	28/08/2017
Aprovação da Indicação de Assessor ad-hoc Concluída - Relatório Científico 1	24/08/2017
Parecer de Assessor ad-hoc Emitido - Relatório Científico 1	11/08/2017
Solicitação enviada a Assessor ad-hoc para emissão de parecer - Relatório Científico 1	08/08/2017
Indicação de Assessor ad-hoc Concluída - Relatório Científico 1	07/08/2017
Indicação de Assessor ad-hoc Cancelada - Relatório Científico 1	21/06/2017
Solicitação enviada a Assessor ad-hoc para emissão de parecer - Relatório Científico 1	03/05/2017
Habilitação Concluída - Relatório Científico 1	03/05/2017
Submissão - Relatório Científico 1	02/05/2017
Assinatura da FAPESP Registrada - Contrato Inicial	13/04/2016
Assinatura do Outorgado Registrada - Contrato Inicial	13/04/2016
Análise da Minuta Concluída - Contrato Inicial	06/04/2016
Preparação da Minuta Concluída - Contrato Inicial	31/03/2016
Verificação da habilitação com resultado "Habilitado" - Contrato Inicial	31/03/2016
Aceite da Concessão com resultado "Aprovado"	22/03/2016
Resultado de Despacho Científico Divulgado - Reconsideração	22/03/2016
Emissão de Despacho Científico Concluída - Reconsideração	21/03/2016
Preparação de Despacho Científico Concluída - Reconsideração	21/03/2016
Pré-Preparação de Despacho Científico Concluída - Reconsideração	15/03/2016
Despacho Científico Iniciado - Reconsideração	15/03/2016
Recomendação da Coordenação Adjunta Concluída - Reconsideração	14/03/2016
Recomendação da Coordenação de Área/Programa Concluída - Reconsideração	07/03/2016
Indicação de Assessor ad-hoc Concluída - Reconsideração	07/03/2016
Habilitação Iniciada - Reconsideração	26/02/2016
Submissão da Solicitação - Reconsideração	11/02/2016
Processo devolvido	02/02/2016
Habilitação Iniciada - Reconsideração	02/02/2016
Submissão da Solicitação - Reconsideração	21/12/2015
Resultado de Despacho Científico Divulgado - Reconsideração	18/12/2015
Emissão de Despacho Científico Concluída - Reconsideração	18/12/2015
Preparação de Despacho Científico Concluída - Reconsideração	16/12/2015
Pré-Preparação de Despacho Científico Concluída - Reconsideração	15/12/2015
Despacho Científico Iniciado - Reconsideração	15/12/2015
Recomendação da Coordenação Adjunta Concluída - Reconsideração	14/12/2015
Recomendação da Coordenação de Área/Programa Concluída - Reconsideração	14/12/2015
Parecer de Assessor ad-hoc Emitido - Reconsideração	23/11/2015
Solicitação enviada a Assessor ad-hoc para emissão de parecer - Reconsideração	27/10/2015

Indicação de Assessor ad-hoc Concluída - Reconsideração	26/10/2015
Habilitação Iniciada - Reconsideração	06/10/2015
Submissão da Solicitação - Reconsideração	05/10/2015
Resultado de Despacho Científico Divulgado - Proposta Inicial	04/10/2015
Emissão de Despacho Científico Concluída - Proposta Inicial	03/10/2015
Preparação de Despacho Científico Concluída - Proposta Inicial	02/10/2015
Pré-Preparação de Despacho Científico Concluída - Proposta Inicial	29/09/2015
Despacho Científico Iniciado - Proposta Inicial	29/09/2015
Recomendação da Coordenação Adjunta Concluída - Proposta Inicial	28/09/2015
Recomendação da Coordenação de Área/Programa Concluída - Proposta Inicial	14/09/2015
Parecer de Assessor ad-hoc Emitido - Proposta Inicial	18/08/2015
Solicitação enviada a Assessor ad-hoc para emissão de parecer - Proposta Inicial	21/07/2015
Indicação de Assessor ad-hoc Concluída - Proposta Inicial	20/07/2015
Habilitação Concluída - Proposta Inicial	06/07/2015
Submissão da Solicitação - Proposta Inicial	25/06/2015
